# A Neural Model of Distance-Dependent Percept of Object Size Constancy

**DOI:** 10.1371/journal.pone.0129377

**Published:** 2015-07-01

**Authors:** Jiehui Qian, Arash Yazdanbakhsh

**Affiliations:** 1 Department of Psychology, Sun Yat-Sen University, Guangzhou, China; 2 Department of Psychological & Brain Sciences, Center for Computational Neuroscience and Neural Technology, Boston University, Boston, Massachusetts, United States of America; Centre de Neuroscience Cognitive, FRANCE

## Abstract

Size constancy is one of the well-known visual phenomena that demonstrates perceptual stability to account for the effect of viewing distance on retinal image size. Although theories involving distance scaling to achieve size constancy have flourished based on psychophysical studies, its underlying neural mechanisms remain unknown. Single cell recordings show that distance-dependent size tuned cells are common along the ventral stream, originating from V1, V2, and V4 leading to IT. In addition, recent research employing fMRI demonstrates that an object’s perceived size, associated with its perceived egocentric distance, modulates its retinotopic representation in V1. These results suggest that V1 contributes to size constancy, and its activity is possibly regulated by feedback of distance information from other brain areas. Here, we propose a neural model based on these findings. First, we construct an egocentric distance map in LIP by integrating horizontal disparity and vergence through gain-modulated MT neurons. Second, LIP neurons send modulatory feedback of distance information to size tuned cells in V1, resulting in a spread of V1 cortical activity. This process provides V1 with distance-dependent size representations. The model supports that size constancy is preserved by scaling retinal image size to compensate for changes in perceived distance, and suggests a possible neural circuit capable of implementing this process.

## Introduction

Humans make stable judgments about an object’s actual size despite changes in its retinal size with distance, which is known as size constancy phenomenon. Previous research proposed a size-distance invariance hypothesis (SDIH) to explain the size constancy. It states that some function of retinal size combines multiplicatively with perceived distance to obtain the perceived size of an object [1–4] Many size illusions, such as the Ponzo and moon illusions, are suggested to be based on this size–distance relationship [5–9]. The SDIH has long been proposed, however, its underlying neural mechanisms remain unclear.

Single cell recordings in awake and anesthetized monkeys confirm the existence of distance-dependent size tuned cells along the ventral pathway from visual cortical area V1, V2 and V4 [10] leading to inferotemporal (IT) cortex [11]. In particular, Dobbins et al. [10] found that a large number of cells in V1, V2, and V4 preferred the same retinal image size, but varied their firing rates with different viewing distances. Among these cells, some showed a monotonic increase in mean firing rate with decreasing distance (nearness cells), some with increasing distance (farness cells), and a few that are distance-independent. These results suggest that size tuned cells whose activities are scaled by distance are common in the visual cortex. In addition, recent functional magnetic resonance imaging (fMRI) studies [12–14] demonstrate that an object’s perceived size modulates its cortical retinotopic representation. A distant object would appear to be larger than a closer object with the same retinal image size. Surprisingly, the apparently larger object causes activation at a more eccentric locus in the primary visual cortex, compared to the apparently smaller object. In other words, the same visual angle projected on the retina could occupy different proportions of V1, depending on the perceived size of the corresponding object. Although these findings contradict the traditional view that retinotopic mapping in V1 is precise and hardwired, emerging evidence supports that visual processing in V1 depends on both retinal image and distance information [15]. The distance may be signaled by feedback of three-dimensional (3D) spatial representation from other brain regions, such as the lateral intraparietal cortex (LIP) [16].

**Fig 1:**
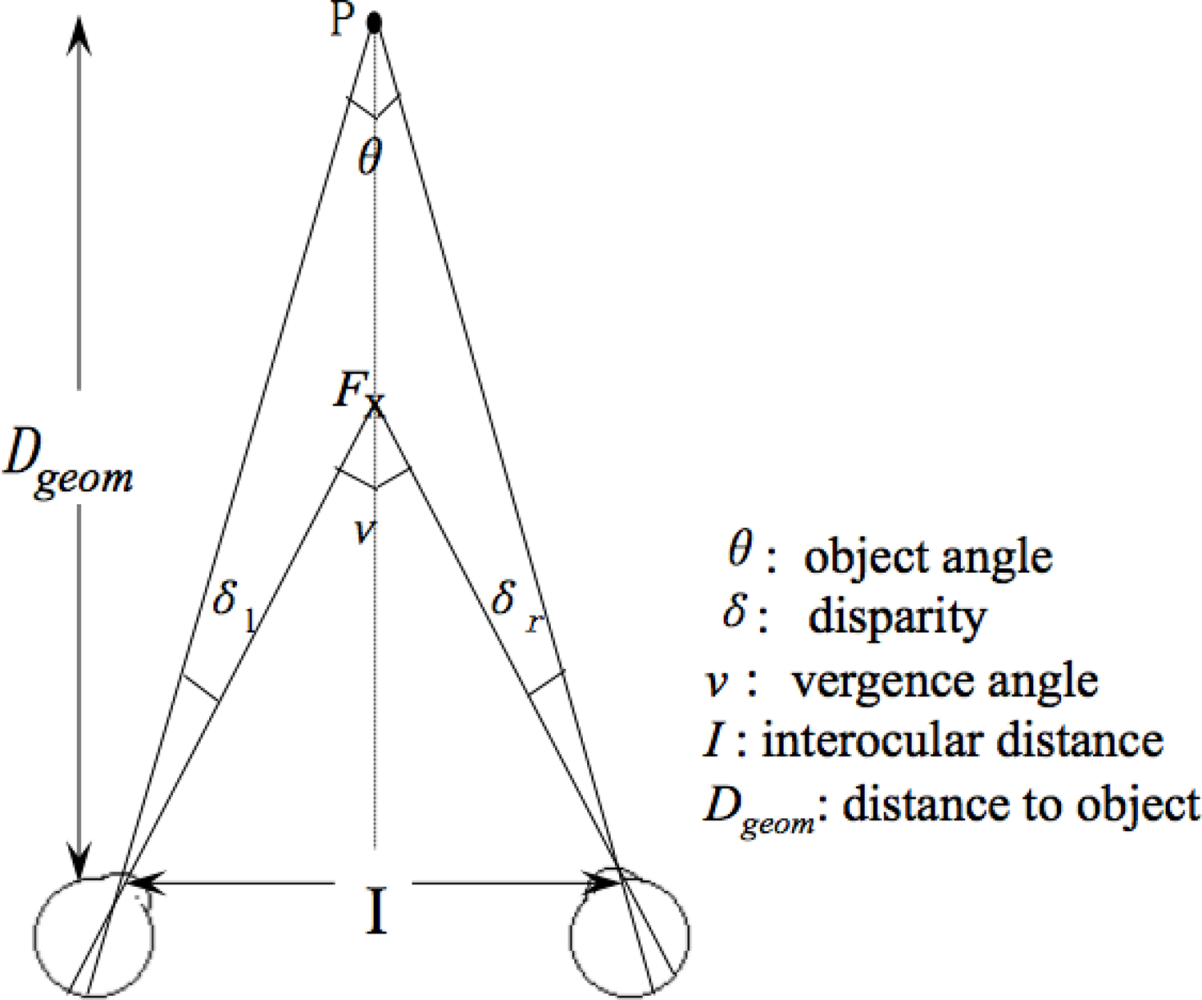
Geometry for stereopsis. ”F” marks the fixation point;”P” marks the position of an object. *D*_*geom*_, indicates the distance from object, and I indicates interocular distance. The horizontal disparity, *δ*, is equal to the difference of the vergence angle, *v*, and the object angle, *θ*. *δ = δ*_*r*_ *+ δ*_*l*_, where *δ*_*r*_ and *δ*_*l*_ correspond to the disparity on the left and right retina respectively.

Distance estimation is crucial to size constancy. Egocentric distance, i.e., the distance of an object from an observer, can be estimated by horizontal disparity (HD) combined with viewing distance. Viewing distance usually refers to the distance to the fixation point. It can be recovered by depth cues, such as vergence angle and vertical disparities [17, 18]. Besides disparity and vergence, there are many other depth cues, such as motion parallax, occlusion, familiar size, and linear perspective, which could contribute to distance perception. However, given the limited viewing conditions used in the single cell and imaging studies, it is assumed that disparity and vergence play a dominant role in distance perception. In this article, we only focus on these two depth cues. If an object is directly in front of the observer, its distance, *D*_*geom*_, can be recovered by a function of HD, *δ*, vergence, *v*, and interocular distance, *I* (see S1 Table for the full list of variables). According to the geometrical layout in Fig 1, the equation is as follows:

$$D_{geom}=\frac{I}{2\text{tan}\left(\frac{v-\delta }{2}\right)}$$ (1)

where *I* normally range from 6 mm to 7 mm. When disparity increases, Eq (1) initially underestimates and then overestimates the perceived distance [19–20]. By modifying *δ*, Eq (1) can approximate perceived distance quite accurately [21]. This modified *δ* is called corrected disparity, see S1 File for details.

Estimation of perceived distance depends on disparity, while disparity estimation may also depend on distance. Many studies have shown that disparity tuned cells are modulated by viewing distance. Trotter et al. [22, 23] found that in alert, behaving monkeys, the responses of a large majority of disparity-tuned neurons in V1 were distance-dependent. They suggested that extraretinal signals, probably vergence or accommodation, could be integrated with disparity early in the visual processing pathways for 3D spatial representation. Other studies supported that vergence can be used as a reliable cue for distance perception [24–26]. Since it is known that cells in V1 are sensitive to HD [27–30], disparity signals in our proposed neural model arise from V1. While studies have suggested multiple loci in the brain responsible for vergence control [31–33], we select the frontal eye fields (FEF) to provide vergence signal in our model [34, 35]. These two depth cues give rise to distance perception. We predict that area MT may be responsible for integrating vergence and disparity signals. Although so far we lack direct neurophysiological evidence to confirm this point, monosynaptic connections have long been found from V1 to MT [36, 37] and FEF to MT [35].

Single cell recordings found that LIP provides a distributed representation of egocentric space [16, 38–40]. It seems that LIP receives inputs from MT and MST, generating a 3D spatial representation that acts as a premotor signal for directing saccades [16]. Based on these finding, we hypotheses that MT feeds the integrated depth information forward to LIP, where a distance map is constructed. Then, LIP feeds the distance information back to MT [39, 41], and even further back to V1, where it regulates responses of size tuned cells to achieve a distance-dependent size representation.

**Fig 2:**
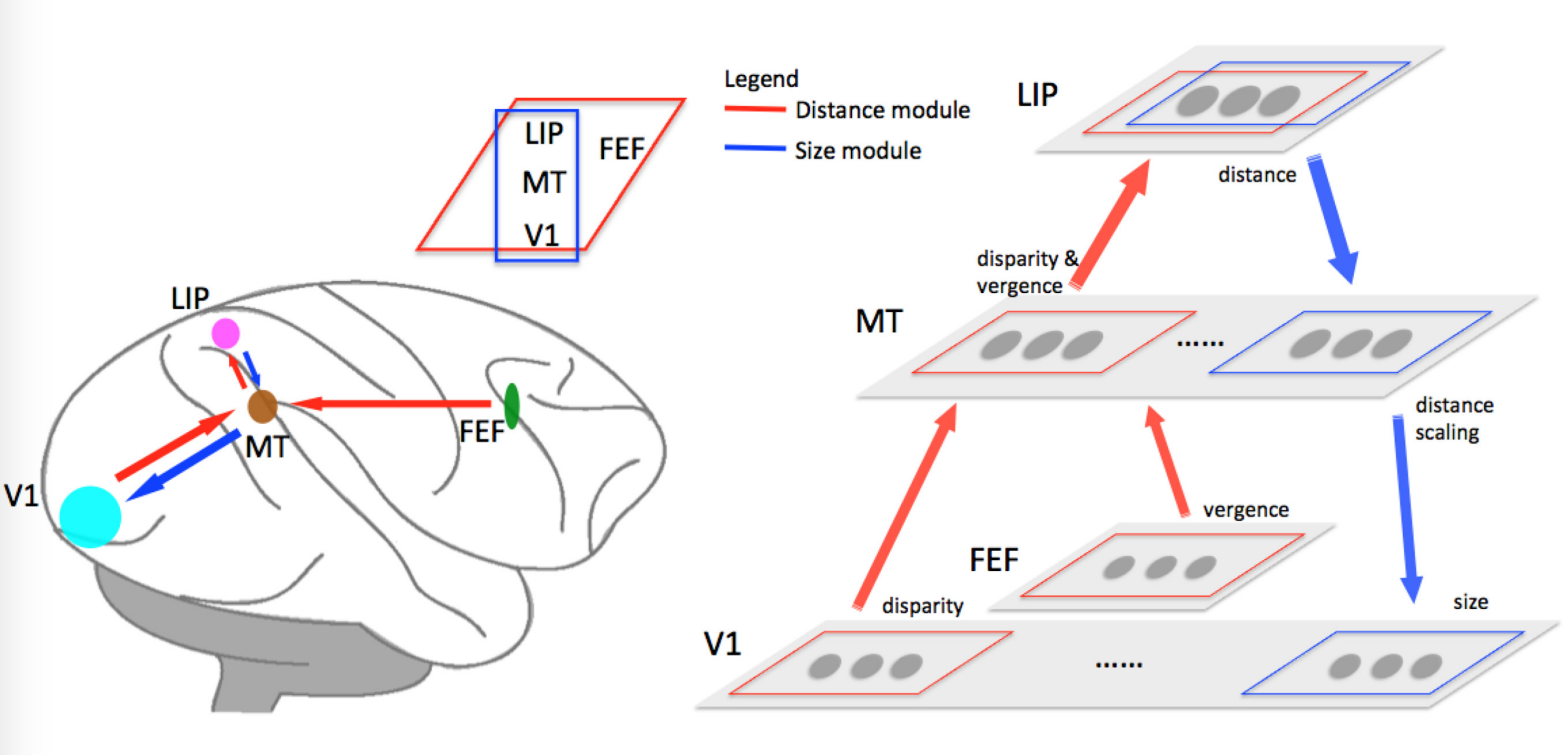
Proposed brain areas and their connections for distance-dependent size perception. Red arrows indicate pathways involved in distance perception: fibers carrying signals from V1 (disparity) and FEF (vergence) terminate in MT; MT then projects to LIP, where a map of distance information is constructed. Blue arrows indicate pathways involved in size perception: the distance information from LIP provides feedback signals to MT, which further projects to V1, modulating neural responses of size tuned cells and cortical activity spread in V1. Thus, a distance-dependent size representation is achieved.

We propose a neural model that simulates the results from Dobbins et al. [10] and Sperandio et al. [14]. The model comprises of a distance module and a size module (Fig 2). In the distance module, first a horizontal disparity signal arises from V1 and vergence signal arises from FEF; then MT neurons gate disparity tuning by vergence; finally, the information in MT neurons pass on to LIP to construct a 3D spatial representation. In the size module, the distance information obtained from LIP provides a feedback signal to gain modulate neural responses of size tuned cells and the spread of cortical activity in V1. Table 1 summarizes the brain areas involved in our model and their proposed functions. However, these proposed functions need to be tested.

**Table 1:** Brain areas and their proposed functions in the model.

**Model areas**	***Distance module* (Ref.)**	***Size module* (Ref.)**
****V1****	disparity coding [22]	size coding [10, 14]
****FEF****	vergence coding [34]	—
****MT****	disparity & vergence (prediction)	distance scaling (prediction)
****LIP****	distance coding [32]	distance

Although there are neural models proposed to simulate the cortical representation of egocentric distance [21], no previous modeling work has clearly demonstrated the neural mechanisms of size constancy. Here, our model simulates the neural responses of distance-dependent size tuned cells in V1, based on the current findings of the neurophysiology and fMRI studies. Our model supports that size constancy could be preserved by scaling retinal image size to compensate for changes in viewing distance, and suggests a neural circuit of how this process is achieved.

## Methods

### Model overview

Our model consists of two sequential stages: first, the distance module where a distance map is created; second, the size module where size tuning is regulated by distance. In the first stage (Fig 2, red arrow), cells in area V1 code for HD using Gaussian functions, while cells in area FEF code for vergence using sigmoidal functions. MT cells integrate the outputs from both V1 and FEF by means of a set of basis functions; the outputs of MT cells feed forward to cells in LIP to construct a distance map. In the second stage (Fig 2, blue arrow), the distance information from LIP feeds back to MT for distance scaling and then back to V1, modulating the activity of size tuned cells. A size representation based on distance therefore can be constructed.

### Distance Module

**Fig 3:**
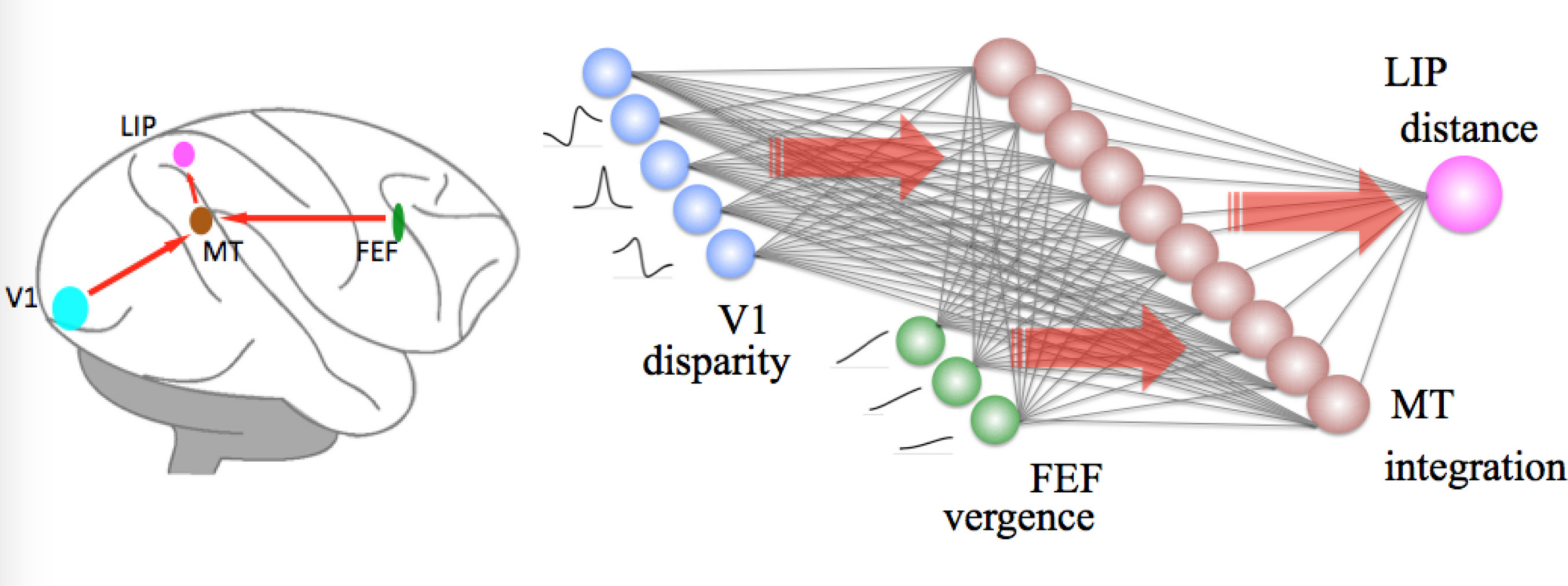
Distance module. Disparity selective cells (see variable a in Eqs (4)–(6)) in V1 are gain-modulated and simulated by Gaussian functions. Vergence selective cells (see variable *v* in Eq (7)) in FEF are simulated by sigmoidal functions. At the level of MT (see variable *B* in Eq (3)), vergence and disparity are integrated. At the level of LIP, a distance map (see variable *D* in Eq (2)) is estimated from a linear combination of MT outputs.

We propose that the distance module consists of bidirectional connections from V1 and FEF to MT and further to LIP (Fig 3). Area MT can be deemed as a layer of processing units integrating inputs of disparity signals from V1 and vergence signals from FEF. It serves as an intermediate transformation between the inputs from V1 and FEF, and outputs to LIP.

The final output of the distance module is a distance map, *D(δ*, *v)*, in LIP. It is estimated from a linear combination of the outputs of MT:

$$D=\overset{n}{\underset{i=1}{\sum }}w_{i}B_{i}\left(\delta ,v\right)$$ (2)

where *w*_i_ is the connection weight from each cell in MT to LIP. *B*_*i*_(*δ*,*v*) corresponds to the responses of gain-modulated MT neuron, *i*, which is specified as follows:

$$B_{i}\left(\delta ,v\right)=a_{i}\left(\delta \right)z_{i}\left(v\right)$$ (3)

where *a*_*i*_(*δ*) and *z*_*i*_(*v*) indicate the tuning curves of disparity- and vergence- selective cells, respectively (see S1 Table for the full list of variables). It is known that disparity-selective cells in V1 are gain-modulated, and can be categorized into one of the six cell types [28, 30] (see Fig 9 of [30]): tuned excitatory (TE)/tuned inhibitory (TI) cell, which gives maximal/minimal responses at zero disparity; tuned near (TN)/tuned far (TF) cell, which has similar disparity tuning function as TE cells but peaks at negative/positive disparity; far (FA)/near (NE) cell, which activates over a wide range of positive/negative disparities. The disparity tuning of V1 neurons are generally described using either Gabor [42–44] or Gaussian functions [21, 45]. In our model, the tuning profile of V1 cells are defined by Gaussians for mathematical simplicity: Near/tuned near (with preferred disparity, *δi*, within [-4°, -1.5°]) cells,

$$a_{i}\left(\delta \right)=A_{1}e^{-\;\frac{{\left(\delta -\delta_{i}\right)}^{2}}{\sigma_{i}^{2}}}-\;A_{2}e^{-\;\frac{{\left(\delta -\left(\delta +\sigma_{i}^{2}\right)\right)}^{2}}{\sigma_{i}^{2}}}+A_{3}$$ (4)

Far/tuned far (with preferred disparity, *δi*, within [1.5°, 4°]) cells,

$$a_{i}\left(\delta \right)=A_{1}e^{-\;\frac{{\left(\delta -\delta_{i}\right)}^{2}}{\sigma_{i}^{2}}}-\;A_{2}e^{-\;\frac{{\left(\delta -\left(\delta -\sigma_{i}^{2}\right)\right)}^{2}}{\sigma_{i}^{2}}}+A_{3}$$ (5)

**Fig 4:**
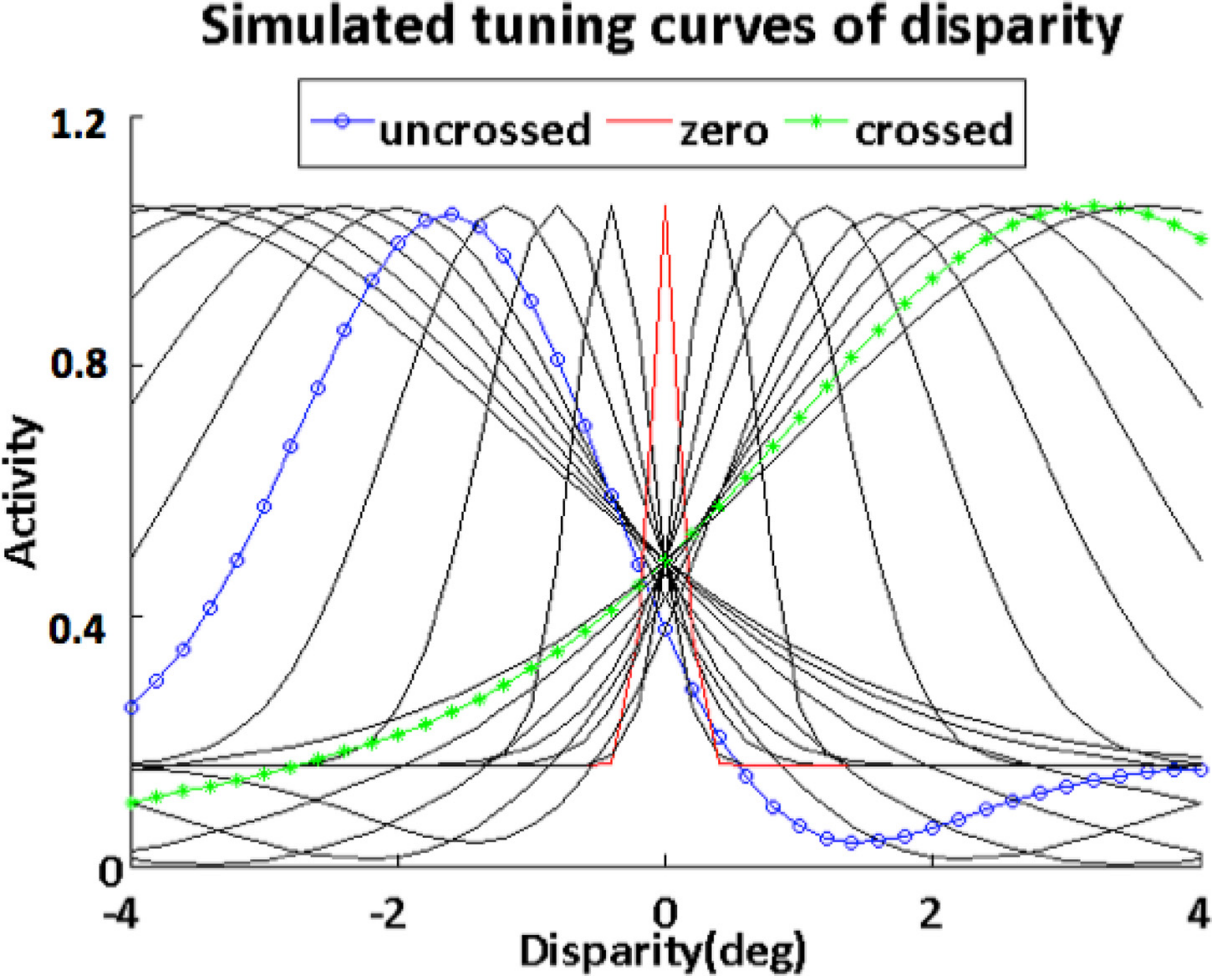
Simulated tuning curves of disparity-selective cells. The cell tuning curves in V1 [28, 30] are simulated by subtracting Gaussians. Each curve corresponds to the response of a single cell and exhibits a similar response pattern as Fig 9 in Gonzalez and Perez [30]. For example, the red dashed line resembles the TE cell’s response, which gives maximal responses at zero disparity; the blue open-circle line resembles the TN cell’s response, which has similar disparity tuning function as TE cells but peaks at negative disparity; the green star line resembles the FA cell’s response, which activates over a wide range of positive disparities. TF and NE cells are simulated but not marked in the figure. TI cells are not included in the simulation, since their responses could be simulated by assigning negative output weights to the TE cells.

Tuned excitatory units (with preferred disparity, *δi*, within [-1.5°, 1.5°]) cells,

$$a_{i}\left(\delta \right)=A_{1}e^{-\;\frac{{\left(\delta -\delta_{i}\right)}^{2}}{\sigma_{i}^{2}}}$$ (6)

where *δi* is the peak of response for a given curve, and *σ*_*i*_ describes the width of the tuning curve for the full list of variables), which is chosen to equal the absolute value of the disparity corresponding to the peak response, except for the curves whose peaks are within the disparity range [-10, 10’], for which *σ*_*i*_ is set to 10 arcmin. Forty disparity tuned cells were assigned. Their tuning curves, *a*_*i*_(*δ*), are shown in Fig 4 (every other cell responses are shown). Resembling Fig 9 in Gonzalez and Perez [30], our model tuning curves exhibit similar response patterns of tuned near, tuned far, tuned excitatory, near and far cells found in primates. Parameters, *A*_1_, *A*_2_, and *A*_3_ were chosen such that each cell types’ tuning curve *a*_*i*_ would have approximately the same maximum value.

**Fig 5:**
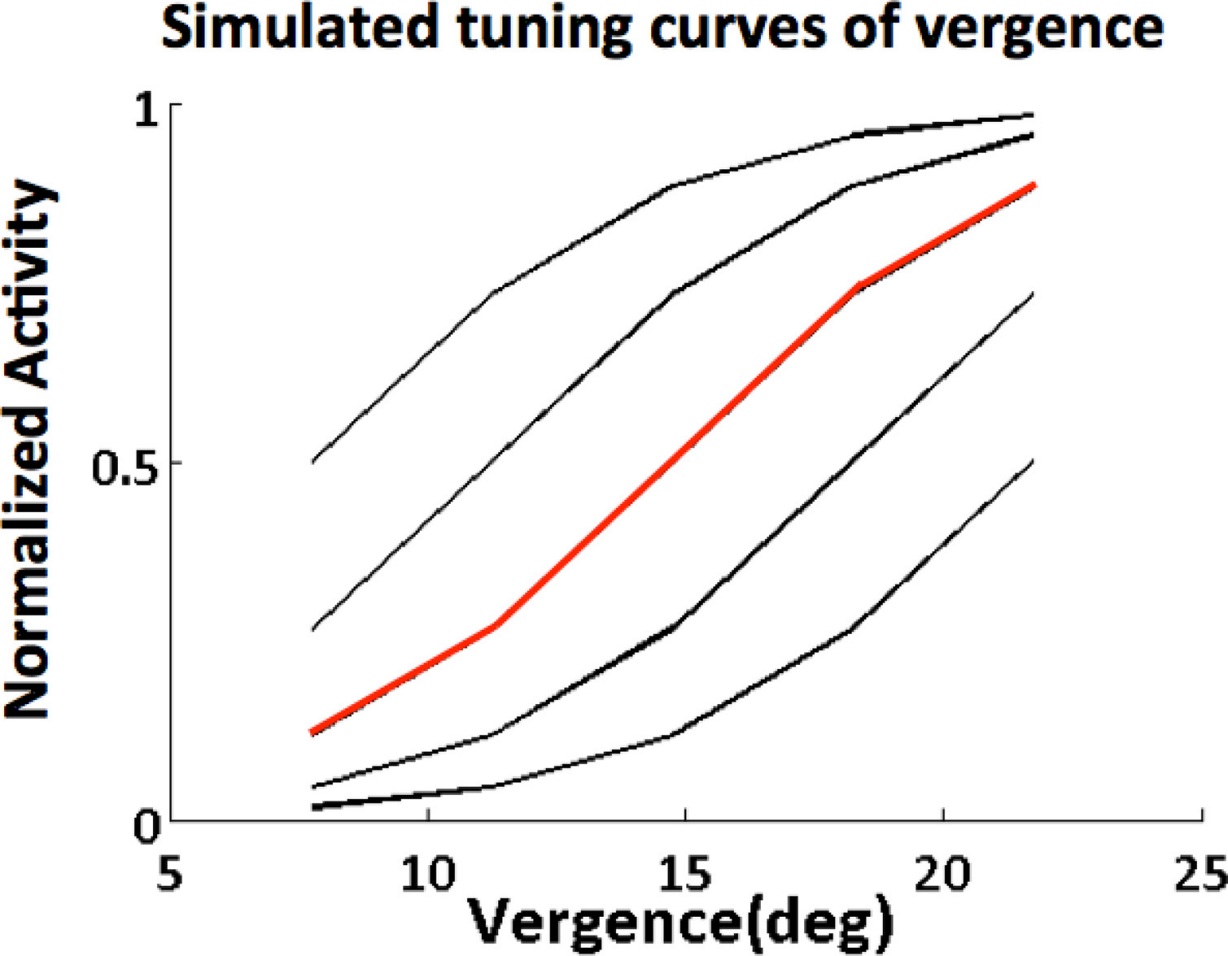
Simulated tuning curves of vergence cells. The modeled tuning curves of multiple vergence cells in FEF are simulated by sigmoids. Each curve corresponds to the response of a single cell to vergence. Similar to Fig 3b in Gamlin [34], our model tuning curves show that cell activity increases as vergence angle increases.

Vergence tuning curves in FEF, *z*_*i*_ *(v)*, are modeled as sigmoids:

$$z_{i}\left(v\right)=\frac{1}{1+e^{-\;\frac{v-v_{i}}{T_{i}}}}$$ (7)

where *v* is the vergence angle, *v*_*i*_ and *T*_*i*_ are the thresholds and the slopes of the sigmoids, respectively. The threshold controls the position of the sigmoid and the vergence axis, and the slope controls the steepness of the sigmoid. We chose five different thresholds, therefore obtaining five vergence tuning curves. The vergence tuning curves are shown in Fig 5. Resembling cell responses recorded from the primate visual cortex (refer to Fig 3b in Gamlin [34]), our model tuning curves show that cell activity increases as vergence angle increases.

Disparity and vergence signals are integrated by a multiplication operator, defined in Eq (3). Since there are forty disparity tuned cells and five vergence tuned cells, we obtain 200 gain-modulated cells in MT. Distance information is estimated in LIP as a linear combination of the weighted outputs from MT. It has been generally assumed that humans accurately perceive geometrical distance based on disparity and vergence. However, people tend to overestimate close viewing distances and underestimate far distances in reality [24], even within the arm’s reaching space [26]. Therefore, we transformed geometrical distance into perceived distance (see S1 File for details). This perceived distance can be a teaching signal to train the network to gain the optimal weights. The delta rule, an iterative optimization technique, is used to find a set of optimal weights for the network.

### Size Module

**Fig 6:**
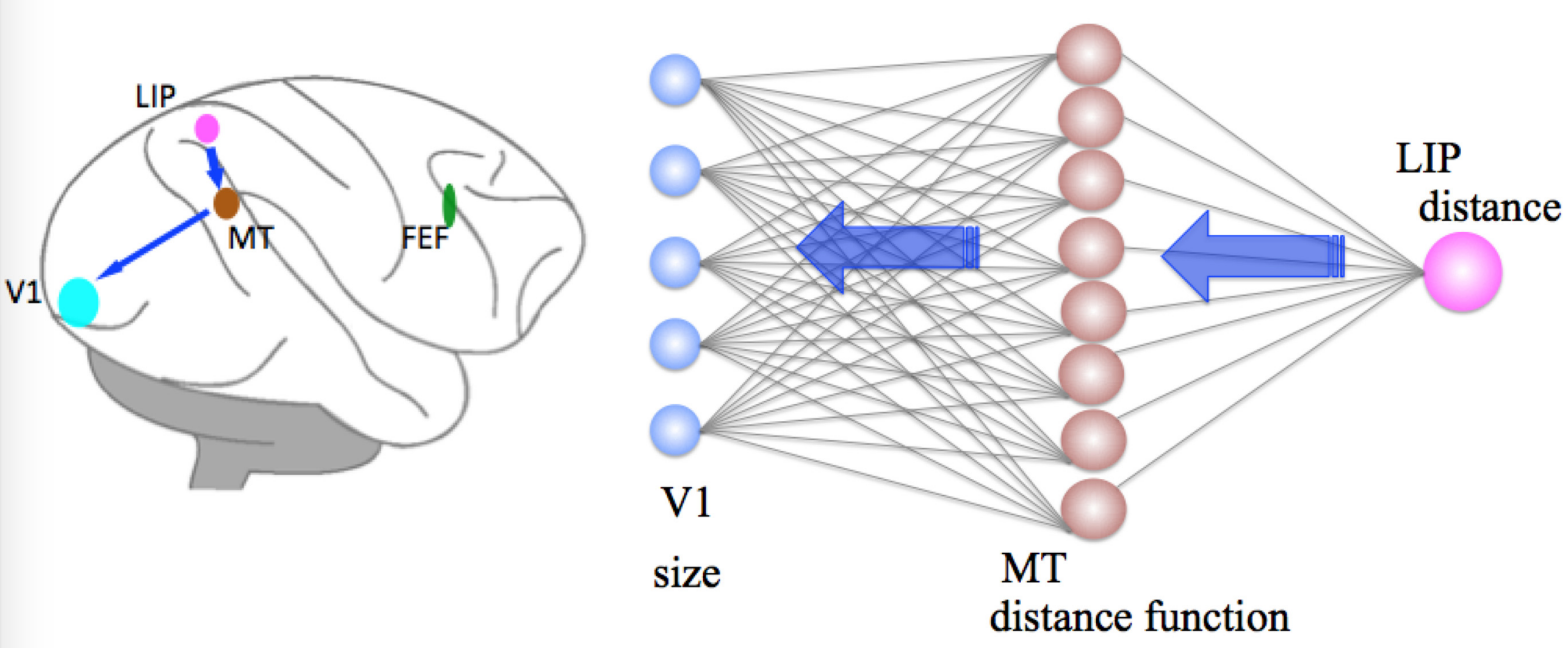
Size module. The distance information (see variable *D*, Eq (2)) in LIP feeds back to size tuned cells (see variable *s*, Eq (8)) in V1 through MT. size tuned cells in V1 are gain-modulated and have Gaussian-shaped tuning curves. In area MT, distance scaling functions are constructed. The activity of the size tuned cells is modulated by distance information through distance-dependent shunting equation (Eq (9)).

The size module consists of feedback connections from LIP to MT and further back to V1 (Fig 6). As we described above, the output of the distance module is a distance map estimated from a set of vergence and disparity input pairs. The distance information feeds back to the size selective cells in V1 through MT, where a distance scaling function is constructed. The distance scaling function modulates the response of size tuned cells in V1. This function can be used to obtain nearness, farness, and distance-independent cells identified in Dobbins et al. [10].

**Fig 7:**
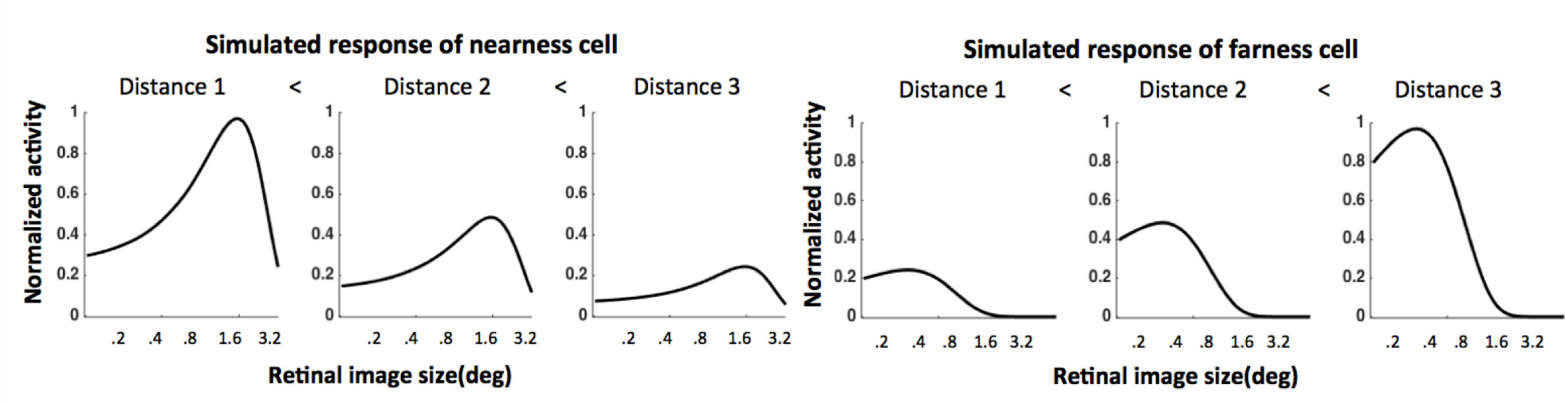
Simulated tuning curves of nearness and farness cells. The cell tuning curves in V1 are simulated by Gaussians. Similar to Fig 1a and 1b in Dobbins et al [10], the magnitudes of the responses of nearness (farness) cells decrease (increase) with viewing distance (Distance 1 < Distance 2 < Distance 3), but the shape and position of the peaks of the tuning curves are similar.

The size-selective cell, *s*_*i*_, is defined by Gaussian:

$$s_{i}=e^{-\;\frac{{\left(\beta -\beta_{i}\right)}^{2}}{\sigma_{i}^{2}}}$$ (8)

where *β*_*i*_ is the peak of response for a given curve, *σ*_*i*_ describes the width of the tuning curve, and *K* indicates a distance scaling factor incorporated by MT cells. *β* is within [0°, 3.2°]. Because there are nearness, farness, and distance-independent cells, different distance scaling functions are set to accommodate the diversity in cell responses. For distance-independent cells, the distance scaling function equals a constant, i.e., *K = C*, cell response does not change over distance. For farness cells, *K = CD(δ*, *v)*, where *D(δ*, *v)* is the distance information obtained from the distance module, cell response increases over distance. For nearness cells, *K = D(δ*,*v)*, cell response decreases over distance. Fig 7 shows that the shape and the position of a simulated nearness/farness cell in response to stimuli with equal retinal image sizes are constant, but the amplitude of the response decrease/increase as the distance increases, just as shown in Fig 1a and 1b in Dobbins’ study.

The model thus far has shown a distance-dependent property of size representation. In addition, in order to reflect size constancy in V1, cortical activity should be relatively constant when viewing an object (with constant physical size) at varying distances. Conversely, when viewing an object with constant angular size at varying distances, the spread of cortical activity in V1 should change [14]. That is to say, with a fixed angular size, the farther the object is, more eccentric parts of V1 get involved. Here, we used a similar stimuli paradigm as in Sperandio et al. to demonstrate the spread of cortical activity: on each trial, stimuli were presented twice (Sperandio et al. used an afterimage instead of a second stimulus presentation). The second stimulus presentation was at one of the three different viewing distances, provoking maximum activation of the corresponding three size tuned cells. In order to demonstrate the cortical activity spread, our model used distance-dependent shunting network equations [46] to simulate the dynamics of change in activity of the size tuned cells. The distance-dependent shunting equation involves basic membrane, or so-called shunting, equation coupled by distance-dependent interactions. Responses of cells provoked by stimulus inputs are regulated by these interactions, and therefore demonstrate distance-related properties. For example, topographic shifts of cell responses in a neural population may be observed in accordance with the spatial shifts of stimulus. In our model, size tuned cells in V1 obey the distance-dependent shunting equations, whereby stimulus input *I*_*k*_ at cell *k* is regulated by Gaussian defined excitatory and inhibitory connections among cells (*s*_*ki*_ and *s’*_*ki*_, where *k ≠ i*). It is critical to simulate the dynamics of cells’ activity, from which we can see the temporal shifts in cell responses affected by distance-dependent interactions. The equation is defined as follows:

$$\frac{dS_{i}}{dt}=-B_{1}S_{i}-\left(B_{2}-S_{i}\right)\overset{n}{\underset{k=1}{\sum }}I_{k}s_{ki}-\left(B_{3}+S_{i}\right)\overset{n}{\underset{k=1}{\sum }}I_{k}s_{ki}^{'}$$ (9)

where *S*_*i*_ is the response of distance-dependent size tuned cell, *i*. In the early visual cortex, most feature-selective cells are organized topographically such that neighboring cells respond to similar quantities of a feature [47]. In our model, we employ the same orderly organization pattern for size-selective cells in V1, so neighboring cells are selective to similar angular sizes. Thus, the excitatory and inhibitory Gaussian kernels are defined by Eq (8). The decay rate of cell activity, *B*_1_, was also regulated by the distance scaling function. In this simulation, *B*_1_ was scaled by *CD(δ*, *v)* to simulate the farness cell response. Parameters *B*_2_ and *B*_3_ are chosen to constrain the permitted values of *S*_i_, *S*_*i*_ > 0.

## Results

**Fig 8:**
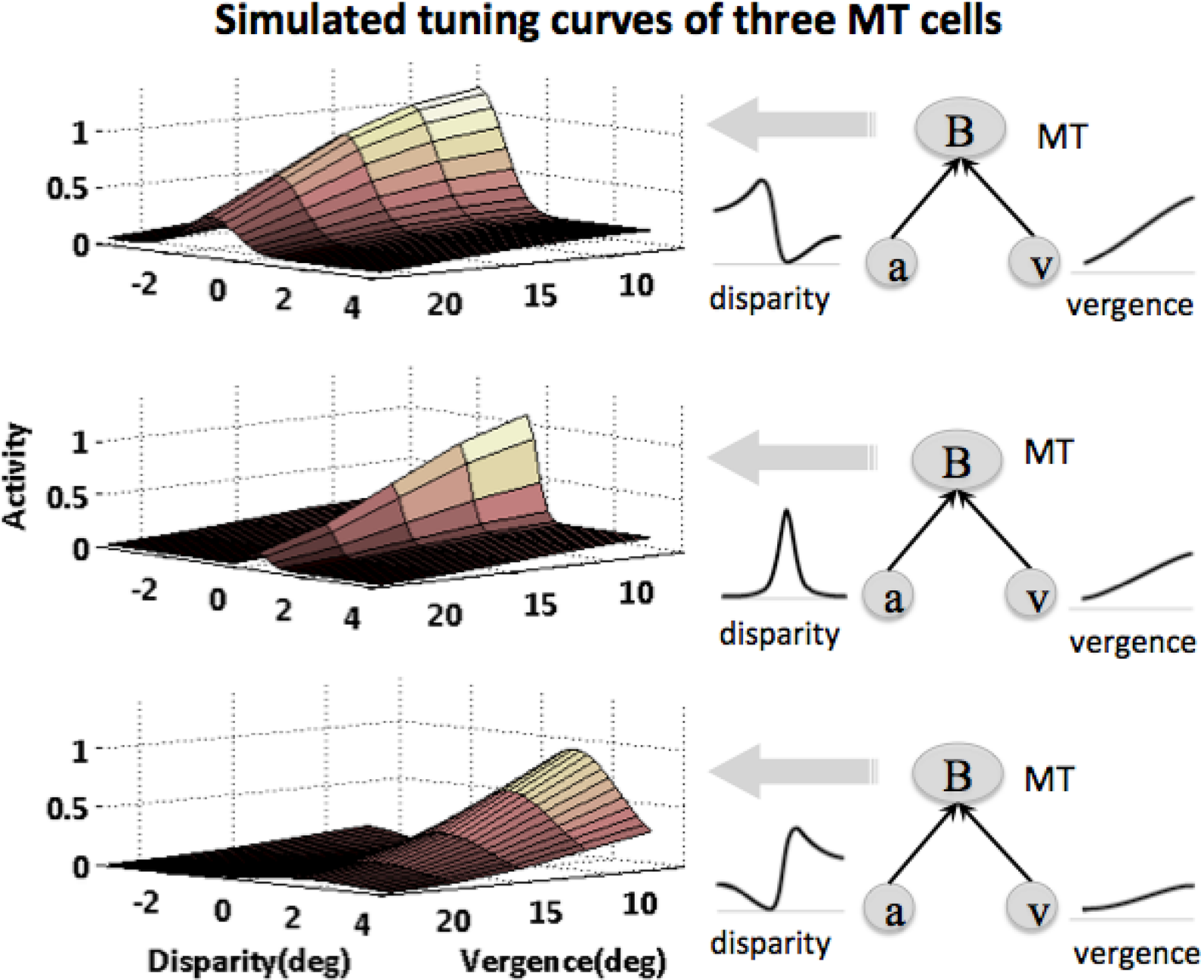
Simulated tuning curves for MT cells in response to vergence and disparity. MT cell responses to disparity is gated by vergence, Three examples of cell activity are shown, corresponding to disparity cells (*a*) tuned to crossed, uncrossed and zero disparity in V1, and vergence cells (*v*) in FEF with different tuning amplitudes.

At the level of MT, disparity and vergence signals are integrated. Fig 8 shows four typical examples of MT cells depicted in the previous section (Eq (3)). Each panel illustrates the simulated activity of a single model MT cell in response to HD and vergence. As shown in the figure, the responses of disparity-selective cells were gated by vergence signals.

**Fig 9:**
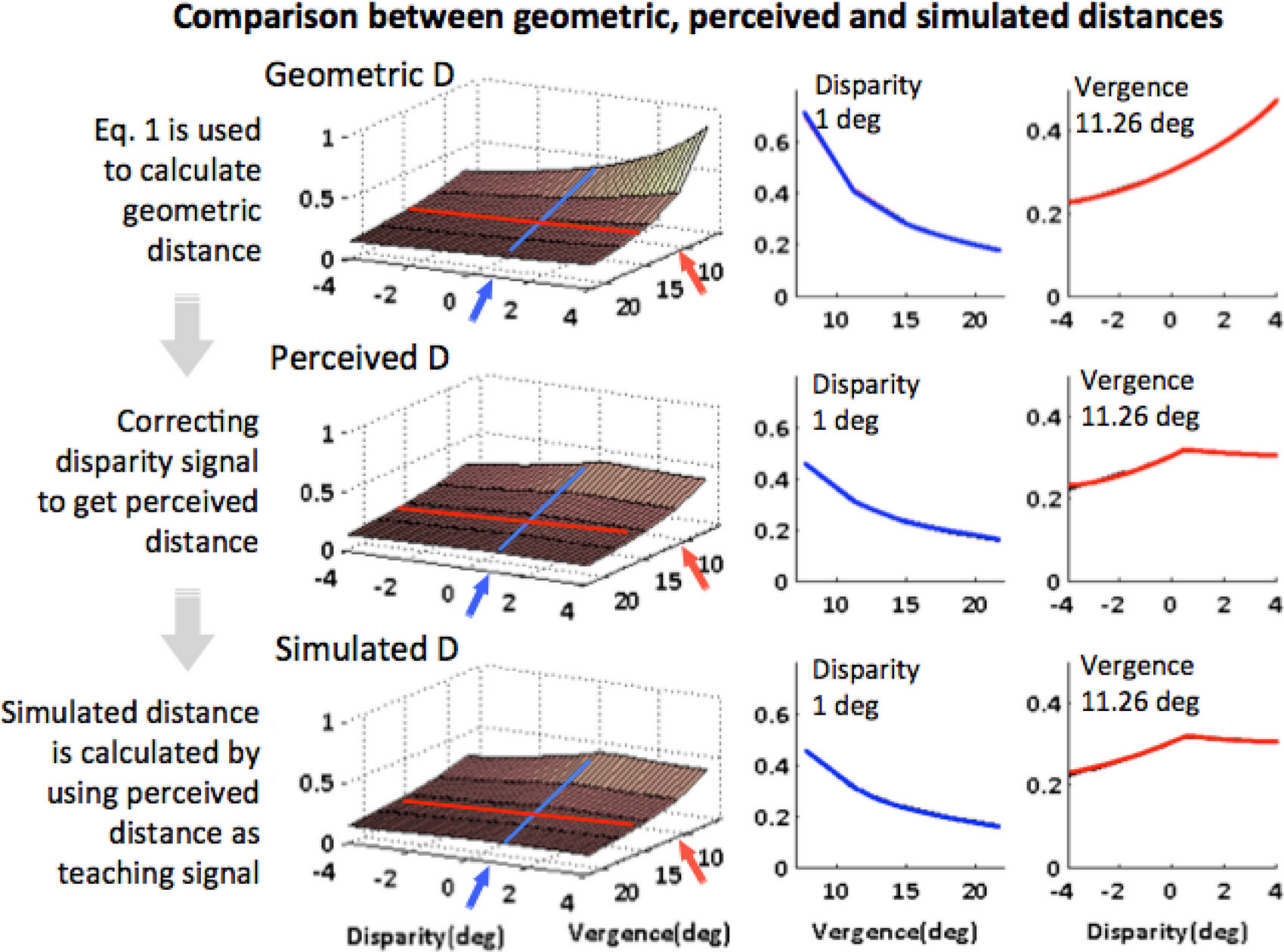
Distance cell response as a function of vergence and disparity in LIP. The top left panel: geometric distance; middle left: approximate perceived distance, calculated by correcting disparity signal in geometric distance (see text and S1 File); bottom left: distance cell response in LIP, simulated by training a network of gain-modulated units of disparity and vergence cells using perceived distance as teaching signal. The right panels show two examples of distance approximation as a function of vergence/disparity, taken from the cross sections (indicated by the red arrows) of the distance response on the left. Y-axes labels for the right panels are the same as of the left ones.

The results of LIP simulation are shown in Fig 9. The top panel depicts geometric distance as a function of vergence and disparity; the middle panel displays the approximate perceived distance; the bottom panel illustrates the approximations by a network of gain modulated units. This distance information output from the model’s LIP then feeds back to MT and to V1 to modulate the activity of size tuned cells.

**Fig 10:**
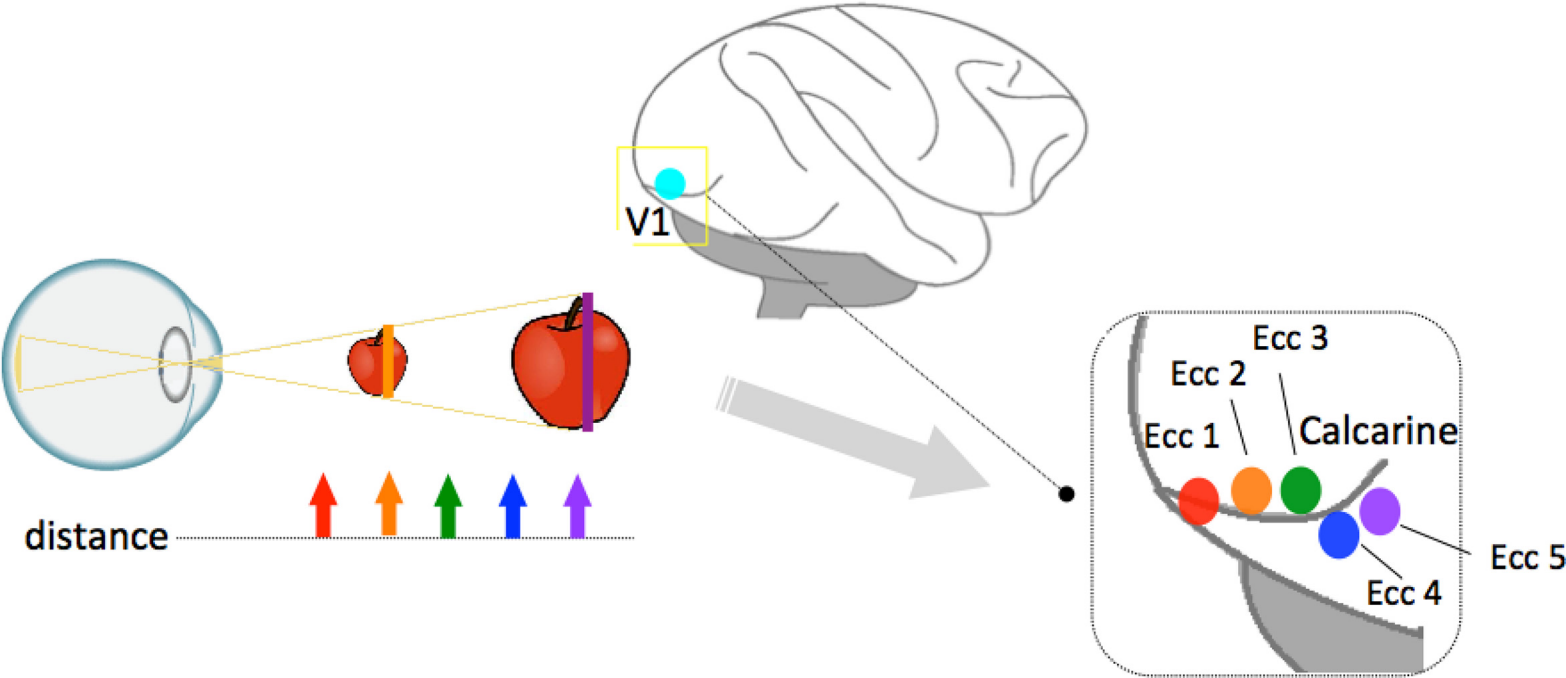
Cortical activity in V1 modulated by perceived size. When a stimulus is presented at a close distance, the activity is strongest in the smaller eccentricity along the calcarine sulcus; when the stimulus with the same angular size (or an afterimage) is presented farther, the activity is stronger in the more eccentric areas. In other words, the bigger the stimuli perceived, the more eccentric the activation in V1. Different colors mark the different eccentric activation corresponding to different viewing distance, e.g., red marks the smallest eccentric activation and purple marks a largest eccentric activation in V1.

**Fig 11:**
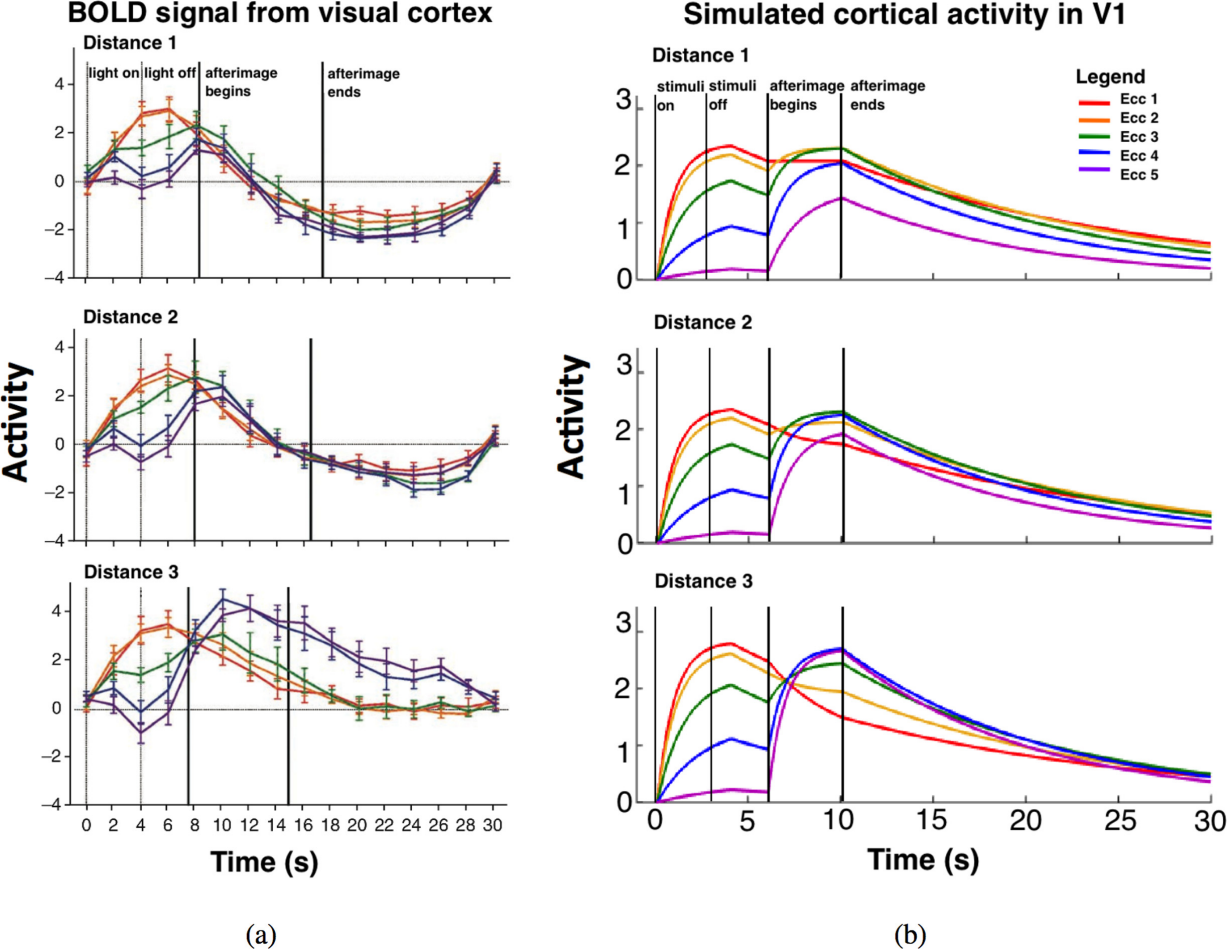
Comparison between experimental data and simulated cortical activity in V1. Panel (a) shows the BOLD signal change recorded from human visual cortex V1 [14]; Panel (b) shows the simulated cortical activity in V1 by our model, which resembles the experimental data on the left. At the beginning of the time courses, when the stimulus is presented at the nearest distance, the activity was strongest in the smaller eccentricity ROIs (marked in red and orange); when the stimulus with the same angular size is presented at a farther distance (one of the three viewing distances tested), the activity is stronger in the more eccentric ROIs (purple and blue) as viewing distance increased. Distance 1 < Distance 2 < Distance 3. Refer to Fig 10 for color marking. Panel (a) is adapted from Sperandio et al. [14].

The cortical activity spread in V1 reported by Sperandio et al. [14] is demonstrated in Figs 10 and 11a. The perceived eccentricity differences are reflected by shifts in the distribution of activity across the surface of V1. Activation in response to perceptually larger stimuli (an afterimage) occurred in a more eccentric position in V1 compared to perceptually smaller stimuli with the same angular size.

Fig 11b shows the simulated cortical activity in V1. As specified in the Methods section, the stimulus was first present at the beginning of the time courses, then it was turn off. After a short interval, the stimulus was turned back on at one of the three different viewing distances, distance 1 < distance 2 < distance 3. Comparing the three panels on the right shows that at the beginning, the activity was strongest at the least eccentric region of interest (Eccentricity 1, marked in red) for all three viewing distances tested, consistent with experimental data from Sperandio et al. [14]. When a stimulus with the same angular size (or an afterimage) was presented at a greater distance, corresponding activity was stronger in the more eccentric region of interest (ROI). For example, compare the bottom panel (distance 3) to the above two panels (distance 1 & distance 2), the ROI related to the largest eccentricity (Ecc 5, marked in purple) gradually increases its activity with viewing distance, and shows the greatest activity at the farthest viewing distance. In other words, the larger the stimuli appeared, the more eccentric the activation in V1.

### Quantitative comparison

We used three measures to compare the quality of the simulation fits to the empirical data: 1) cosine of correlation angle (CCA); 2) difference in root mean square (dRMS); 3) Pearson product-moment correlation coefficient, r.

#### Cosine of correlation angle (CCA)

The CCA is defined as the inner product of the simulation and the BOLD signal curve vectors divided by their norms (see Eq (10)). If the two vectors are parallel with each other, CCA equals 1. The closer it gets to 1, the better the fit is.

$$CCA=\frac{\langle F,S\rangle }{\left|\left|F\right|\left|\cdot \right|\left|S\right|\right|}$$ (10)

where *F* is the vector of the BOLD signal change, and *S* is the vector of the model simulation.

#### Difference in root mean square (dRMS)

Root mean square is anther metric that can be extracted directly from the time-domain signal. It is defined as the square root of the average squared value of the signal and can also be called the normalized energy of the signal:

$$RMS=\sqrt{\frac{1}{n}\;\sum_{i=0}^{n}S_{i}^{2}}$$ (11)

The dRMS is defined as

$$dRMS=\frac{RMS\left(F-S\right)}{RMS\left(F\right)+RMS\left(S\right)}$$ (12)

which shows how much the curves are off from each other, normalized by their own values. The closer it gets to 0, the less the difference is.

#### Pearson product-moment correlation coefficient, r

Pearson’s r is defined as

$$r=\frac{\text{cos}\left(F,S\right)}{\sqrt{var\left(F\right)var\left(S\right)}}$$ (13)

Quantitative comparison shows that 1) the CCA equals .903, with 95% confidence interval (CI) of [.806, 1.000]; 2) the dRMS equals .288, with 95% CI of [.098, .479]; 3) Pearson’s r equals .622, p < .001.

## Discussion

We propose a neural model that achieves distance-dependent size tuning in V1. The model consists of bidirectional connections from V1 and FEF to LIP through MT. A subset of disparity-selective cells in V1 and vergence-selective cells in FEF contribute to the creation of a 3D spatial map in LIP through feedforward interareal connections via MT. The distance information from the 3D representation feeds back to a subset of size tuned cells in V1, modulating their responses and the spread of cortical activity. Our simulation of this model is consistent with neurophysiological findings [10, 14], which suggests that distance-dependent size representation in V1 is a manifestation of size constancy mechanisms.

Under normal viewing conditions, size constancy of humans is nearly perfect for distances under 100 feet [48–50]. Researchers have proposed that this prominent perceptual ability can be achieved by scaling retinal size to compensate for viewing distance. In accordance with a large body of psychophysical evidence [1–4], Dobbins et al.’s neurophysiological findings support this hypothesis, that the magnitudes of size tuned cells’ responses in V1, V2 and V4 are modulated by viewing distance [10]. They suggested that distance-dependent modulation of cell’s responses is possibly a common property of neurons along the ventral visual pathway, from V1 to IT. Apart from size, other features may share this property. For example, disparity tuned cells in V1 show similar activity patterns in response to change in viewing distance [22]. In addition, distance and angle of gaze modulate neural responses in parietal cortex as well as on the dorsal pathway from V1 to parietal cortex [16]. These results suggest that spatial modulation exists in both the dorsal and ventral visual cortical streams, and appears to be a fundamental attribute of the visual cortex.

In order to realize spatial modulation, an estimate of distance is necessary for visual perception so that distance scaling can be applied to all related visuocortical areas. A few neural models have been proposed to address the distance estimation problem. In particular, Pouget et al. [21] proposed a neural network showing that cortical representation of egocentric distance could be computed by combining the outputs from gain-modulated Gaussian units and sigmoids, simulating the responses of disparity- and vergence- selective cells, respectively. Although Pouget et al. did not tackle the problem of size constancy, they mentioned that the neural mechanisms underlying perceptual constancies might adopt a strategy similar to the strategy of distance estimation (gating the activity of disparity-selective neurons by vergence signals). In other words, the activity of certain feature-selective neurons was gated by distance signals to achieve constancy. Other than their study, a few neural models were proposed to address similar topics [51, 52]. But till present, no previous modeling work has explicitly demonstrated the neural mechanism of size constancy.

Our model adopts a similar method, as proposed in Pouget et al., to estimate distance. Distance information modulates the neural activity of size tuned cells, providing distance-dependent size tuning reported by Dobbins et al. [10]. In addition, our model simulates recent findings that demonstrate associations between the spatial extent of activation in human primary visual cortex and an object’s perceived size [12–14]. Activity in V1 becomes more eccentric as the perceived size of a real stimulus, or an afterimage, increases, even though the size of the retinal image remains the same. The retinal signals reaching V1 are modulated by distance information in a fashion that reflects the implementation of size-constancy mechanisms. It is suggested that the spread in cortical location can hardly be explained by local interactions between the stimuli and the 3D scene (e.g., local contrast), because the local interaction would have increased or decreased the overall magnitude of neural activity, rather than induced the cortical activity spread. In addition, attention allocation is required to perform size constancy, which rules out stimulus-based explanations for the spatial spread in activity [13]. Feedback from higher visual areas would seem to make an important contribution to this effect. Specifically, extracting distance information from depth cues, such as linear perspective and texture cues, requires that the information be integrated over a large area with large receptive fields comparable in size to those found in higher visual areas.

In our proposed model, depth cues are integrated along the dorsal pathway leading to LIP. Empirical studies show that LIP may provide egocentric distance representation [16, 38–40]. We further propose that integration of vergence and disparity takes place in MT. Although Chowdhury et al. [53] showed that vertical disparity cues to viewing distance did not modulate MT horizontal disparity tuning, currently no study has directly examined whether the responses of disparity selective cells in MT are gated by vergence signals. Given MT’s anatomical connectivity with LIP, V1, and FEF [35–37], we suggest that it is an appropriate candidate for integrating disparity and vergence. In addition, there are studies showing that the role of MT is not limited to processing moving objects. For example, many MT neurons exhibit robust disparity tuning in response to stationary random-dot patterns [54], and are involved in representing implied motion from static images [55, 56]. On the other hand, in the ventral stream, a possible integration area is V3v. A recent study showed that left V3v may be involved in integrating retinal size and distance information [57]. But to our knowledge, currently no area found along the ventral stream directly codes distance information. Therefore one possibility is that V3v receives distance feedback from LIP via MT and V3A. However, these proposed functions of the brain areas require further investigation and confirmation.

Fig 11b simulates the spread of cortical activity in V1 reported by Sperandio et al. [14]. Quantitative comparison shows that the simulation curve fits the BOLD signal well. Although the overall shape of the simulation and the empirical data matches, there are small differences. The simulated curves have two distinctive phases while the empirical data does not. This can be attributed to the fact that BOLD signal change reflects the activity of a population of neurons, while simulated data reflects activity from a single neuron. For the empirical data, Eccentricity 1–3 showed more activity during the light-on duration (and consequently followed by the light-off duration) than Eccentricity 4&5, because the annuli sizes of the ROI localizers for Ecc 1–3 were close to the light size used to induce the afterimage (in terms of retinal projection). The lack of high amplitude in BOLD response in Ecc 4&5 was followed by a gradual amplitude elevation within the light-off period and continued further during afterimage interval. This indicates that there are time-consuming neural processes going-on to build up and feedback the higher eccentricity parts of V1. These all give the appearance to the empirical data as if in Ecc 4&5 there are no two distinctive phases. Although the positions of peaks of the BOLD signal curves almost stay the same through Distance 1 to 3, the higher-eccentricity parts of V1 in response to the afterimages viewed at larger distances (and hence perceptually larger) seemed to have delayed peaks (as if there is a right peak shift). This delayed BOLD amplitude elevation could also be due to the time-consuming neural processes in bringing activities back to higher-eccentricity parts in V1. In our simulation, there was a surge of activities at the start of the afterimage, and this rebound of activity gave the bi-phasic appearance to the simulated curves. This is that the simulation shows cell response, while the fMRI data is”low-pass filtered” in time domain and hence the rebound of activity could be smoothed out. Besides this, other aspects are consistent with the fMRI data.

The simulation results were achieved by first modulating the responses of size tuned cells in V1 with distance scaling, which is derived in MT through feedback from LIP. Second, our model of V1 is topographically organized by size selectivity; this allows spreading of cortical activation when using distance-dependent shunting equations to simulate the dynamics of cell activity. The result supports that the topographic representation in V1 is dependent on the egocentric distance estimation. The model predicts that the neurons classical receptive fields shift to more eccentric cortical locus. As the perceived distance increases, more neurons are activated, therefore shows a spatial spread of cortical activity. Moreover, in order to simulate the response pattern seen in Fig 11a, distance feedback needed to modulate the activities of V1 farness cells in the simulation. Therefore, the model predicts that the type of size-selective cell (see Fig 7) affects the size of neurons population receptive fields. For example, when an object gets farther away, the farness cells get more involved and the size of their population RF increases. In addition, an important implication of the model is that the spatial spread of cortical activity may be found in other brain areas where cells show topographic organization, and where their activity (possibly in response to features other than size) is affected by distance. The distance information, once extracted, can be used to rescale retinotopic representations in other visual areas. It is possible that the cortical activity spread is a neural marker coding for changes in viewing distance. This prediction can be tested in neurophysiological experiments that may further shed light on the neural mechanisms of distance-related feature perception. For example, neurophysiological methods could assess whether cells selective for curvature or contrast demonstrate similar response properties (like dependent spreading of cortical activation). It has been shown that perceptual constancies may be intercorrelated, e.g, size and contrast constancy, and it is possible that these phenomena share a common scaling factor [8, 9].

In our model, disparity and vergence are the only two depth cues used. In addition to these two cues, numerous psychophysical studies have shown that the vertical disparity (VD) can provide viewing distance information independent of vergence [58–60]. Rogers & Bradshaw [18] showed that it can be used to judge absolute distance and disparity scaling. Durand et al. [61, 62] found that VD encoding shared a common neural process with the HD encoding, with narrower widths of the tuning curves and narrower range for VD. The range extended to a larger eccentric angular scale in parafoveal V1, while centered on a VD value of 0° in foveal V1[63]. This discrepancy might due to the fact that VDs do not naturally occur in central vision, typically for objects smaller than 20° [18, 64]. Although VD is unlikely to be effective in specific experimental situations that our model addressed, it can be easily integrated into our model. Since egocentric distance is usually constrained in laboratory settings, it is reasonable to assume that disparity and vergence are the dominant depth cues in our simulation.

In the real world, distance can be infinitely far where disparity and vergence cues become less effective if not absolutely ineffective. Many other depth cues may be used, like pictorial and motion-related depth cues. Although not conventionally deemed as depth cues, looming or optic flow, which provides information about an observer’s self-motion, could be integrated to estimate distance travelled as well. Therefore, other depth cues may gradually take over the dominant role and contribute significantly in distance estimation. For instance, one computational model has tackled the problem of time to contact by integrating disparity and looming cues, weighted by stimulus size [53]. Estimating time to contact shares some common factors with estimating distance, like involving integration of different depth cues. In principle, the more coherent depth cues can be utilized, the more accurate the distance estimate. It is therefore important for a neural model to have compatibility. Our model can accommodate such situations, e.g., vertical disparity, optic flow, etc. Vertical disparity can be included by extension in the input representation. Besides disparity and vergence, it can be a third variable used in the basis function set to estimate distance. As for optic flow, it is known that neurons in MST are selective for its direction and speed [65]. With cortical connections from MST to MT [66], depth information extracted from optic flow can feed MT, where it integrates other depth cues.

The visual areas used in our model are by no means exhaustive, and it is extremely likely that many other visual areas are involved. Further research may shed light on other possible brain areas contributing to constancy mechanisms. Investigating these questions may clarify how distance information can be estimated or used to modulate cell activity, and therefore improve our understanding of perceptual constancy phenomena.

## Conclusions

Recent research has shown that the activity of size tuned cells in V1 is possibly regulated by feedback of distance information from other brain areas, demonstrating size constancy. We propose a physiologically based neural model that simulates activity of distance-dependent size tuned cells in V1 [10, 14]. The model suggests that many brain areas may share a common set of distance scaling functions, which is essential for three-dimensional spatial coding in visual processing. In addition, we speculate that the topographic spread of cortical activity may be found in other brain areas whose activity (possibly in response to features other than size, like contrast) is modulated by distance information.

## Supporting Information

S1 File Training algorithm. (DOCX)

S1 Table Variables and their definitions. (DOCX)
