# Supplementary material for: A Neural Model of Distance-Dependent Percept of Object Size Constancy
Source: PLoS One. 2015 Jul 1;10(7):e0129377. doi: 10.1371/journal.pone.0129377 (PMC4489391; doi:10.1371/journal.pone.0129377)
Supplement: S1 File — (DOCX) [file pone.0129377.s001.docx]

**Supporting Information**

**S1 File. Training algorithm.**

The perceived distance can be used as teacher signals. The perceived distance function can be obtained by replacing geometric disparity, *δ*, with corrected disparity *δ_c_* in geometric calculation of the distance, Eq. (1). It is because that the psychometric curve of corrected disparity as a function of geometric disparity is nonlinear. The perception of depth appears to be accurate within the disparity range of [- 40’, 40’], but with very extreme disparities, depth appears to fall back to zero, because of the physiological limits for disparity detection. Overall, perceived depth appears to increase and then decrease with disparity, resulting an inverted U-shaped curve. The corrected disparity is defined as follows:

$$\delta'=1.8\delta e^{-{|1.5\delta|}^{0.9}}$$

$$\delta_{c}=\{\begin{matrix} \delta if \delta>\delta' \\ \delta^{'} otherwise \end{matrix}$$

where *δ’* indicates the threshold at which geometric disparity is replaced with corrected disparity.

The optimum weights are the ones that minimize the mean square error:

$$e=\frac{1}{2}\sum_{p=1}^{m} {(y_{p}-\sum_{i=1}^{n} w_{i}B_{i}(\delta_{p}, v_{p}))}^{2}$$

where *m* is the number of examples, *y_p_* is the value of the target for an input vergence and disparity pair, *(δ_p_, v_p_)*. *B_i_(δ_p_, v_p_)* is the value of the MT cell *i* for example *p*, and *w_i_* is the weight or coefficient associated with MT cell *i*. The delta rule changes the weights by a small amount in direction opposite to the gradient of increasing error:

$$\Delta w_{i}= =-\boldsymbol{\alpha}\frac{\partial e}{\partial w_{i}}$$

$$\Delta w_{i}= \boldsymbol{\alpha}\sum_{p=1}^{m} \left( y_{p}-\sum_{i=1}^{n} w_{i}B_{i}(\delta_{p}, v_{p}) \right)B_{i}(\delta_{p}, v_{p})$$

where $\boldsymbol{\alpha}$ is a parameter that controls the rate at which the weights change, *y_p_* is the value of target for an input vergence and disparity, used in the training set. The delta rule can converge to a minimum of the error after a sufficient number of presentations of the training set if $\boldsymbol{\alpha}$ is sufficiently small.
