# Supplementary material for: A Neural Model of Distance-Dependent Percept of Object Size Constancy
Source: PLoS One. 2015 Jul 1;10(7):e0129377. doi: 10.1371/journal.pone.0129377 (PMC4489391; doi:10.1371/journal.pone.0129377)
Supplement: S1 Table — (DOCX) [file pone.0129377.s002.docx]

**Supporting Information**

$${}\begin{matrix} \\ {} \end{matrix}$$

**S1 Table. Variables and their definitions.**

S1 Table lists the definitions of the variables referred in the article.

| Variables | Definitions |
| --- | --- |
| *D* | distance |
| *I* | interocular distance, 6mm |
| *n* | number of cells in MT |
| *T* | slope of the sigmoidal tuning curves of vergence |
| *v* | vergence, degrees of visual angle |
| $\boldsymbol{\sigma}$ | width of a tuning curve of disparity cell |
| *δ* | disparity, degrees of visual angle |
| $\boldsymbol{a}_{\boldsymbol{i}}\left( \boldsymbol{\delta} \right)$ | tuning curve of disparity cell *i* in V1,   *δ* |
| $\boldsymbol{B}_{\boldsymbol{i}}\left( \boldsymbol{\delta,v} \right)$ | MT cell *i* which integrates disparity and vergence (basis function) |
| *s* | tuning curve of size |
| $\boldsymbol{z}_{\boldsymbol{i}}\left( \boldsymbol{\delta} \right)$ | tuning curve of vergence, *v* |
| *m* | number of training examples |
| *e* | mean square error, used in the training set |
| *w_i_* | connection weight from MT cell *i* to distance coding cell in LIP |
| *D_p_* | perceived distance, used for the training set |
| *v_p_* | target vergence, used in the training set |
| *y_p_* | value of target for an input vergence and disparity, used in the training set |
| $\boldsymbol{\alpha}$ | parameter controlling the rate of weight change, used in the training set |
| *δ_c_* | corrected disparity, degrees of visual angle, used in the training set |
